# Selected Molecular Targets for Antiepileptogenesis

**DOI:** 10.3390/ijms22189737

**Published:** 2021-09-08

**Authors:** Marek J. Pawlik, Barbara Miziak, Aleksandra Walczak, Agnieszka Konarzewska, Magdalena Chrościńska-Krawczyk, Jan Albrecht, Stanisław J. Czuczwar

**Affiliations:** 1 Department of Neurotoxicology, Mossakowski Medical Research Institute, Polish Academy of Sciences, 02-106 Warsaw, Poland; mpawlik@imdik.pan.pl; 2 Department of Pathophysiology, Medical University of Lublin, 20-090 Lublin, Poland; barbaramiziak@umlub.pl (B.M.); aleksandrawalczakmd@gmail.com (A.W.); agnkonar@gmail.com (A.K.); 3 Department of Child Neurology, Medical University of Lublin, 20-093 Lublin, Poland; madziachr@wp.pl

**Keywords:** epileptogenesis, antiepileptic drugs, losartan, nonsteroidal anti-inflammatory drugs, antioxidative drugs, antagomirs, c-Fos, epileptogenesis markers

## Abstract

The term epileptogenesis defines the usually durable process of converting normal brain into an epileptic one. The resistance of a significant proportion of patients with epilepsy to the available pharmacotherapy prompted the concept of a causative treatment option consisting in stopping or modifying the progress of epileptogenesis. Most antiepileptic drugs possess only a weak or no antiepileptogenic potential at all, but a few of them appear promising in this regard; these include, for example, eslicarbazepine (a sodium and T-type channel blocker), lamotrigine (a sodium channel blocker and glutamate antagonist) or levetiracetam (a ligand of synaptic vehicle protein SV2A). Among the approved non-antiepileptic drugs, antiepileptogenic potential seems to reside in losartan (a blocker of angiotensin II type 1 receptors), biperiden (an antiparkinsonian drug), nonsteroidal anti-inflammatory drugs, antioxidative drugs and minocycline (a second-generation tetracycline with anti-inflammatory and antioxidant properties). Among other possible antiepileptogenic compounds, antisense nucleotides have been considered, among these an antagomir targeting microRNA-134. The drugs and agents mentioned above have been evaluated in post-status epilepticus models of epileptogenesis, so their preventive efficacy must be verified. Limited clinical data indicate that biperiden in patients with brain injuries is well-tolerated and seems to reduce the incidence of post-traumatic epilepsy. Exceptionally, in this regard, our own original data presented here point to c-Fos as an early seizure duration, but not seizure intensity-related, marker of early epileptogenesis. Further research of reliable markers of early epileptogenesis is definitely needed to improve the process of designing adequate antiepileptogenic therapies.

## 1. Introduction

Antiepileptic drugs (AEDs; also named antiseizure drugs) efficiently control epileptic seizures in no more than ca. 65–70% of patients with epilepsy; this limitation holds for AEDs of the second and third generations as well, which substantiate a search for alternative treatment approaches [1]. Considering further that AEDs are only administered to patients with developed epilepsy, their antiepileptogenic potential has been a matter of dispute [2].

Epileptogenesis is a durable process that converts a normal mammalian brain into an epileptic one [3,4]. It therefore seems very likely that stopping or at least modifying the progress of epileptogenesis may eventually prevent the occurrence of epilepsy.

## 2. Mechanisms of Epileptogenesis

An initial insult, which may comprise status epilepticus, stroke or head trauma, is required to initiate epileptogenesis [3,4]. Interestingly, this process may persist after the onset of seizures and may negatively affect the seizure frequency [4]. Following the initial insult, two subsequent stages may be considered: (i) an acute one lasting from hours to weeks and encompassing neurodegeneration, elevated inflammatory activity and transcriptional events and (ii) a chronic one, taking even months, characterized by a sequence of events typically including neurogenesis, the sprouting of mossy fibers, reorganization of neuronal circuits and gliosis [5]. There is a correlation between mossy fiber sprouting (to the brain locations they never exist in) and the expression of seizure activity. Additionally, dentate granule cells present aberrant locations in the dentate gyrus (so-called ectopic granule cells), forming abnormal connections within the molecular layer of the dentate gyrus and in neurons located in the hippocampal CA3 field, which eventually results in the formation of excitatory circuits [6]. After all, gliosis may participate in the progress of epileptogenesis, as astrocytes may release a number of neurotransmitters and modulators, glutamate being the main excitatory neurotransmitter involved in the generation of epileptiform discharges [5]. In spite of the initial insult (for instance, acute seizure activity) that elevates the number of newborn neurons, neurogenesis within the hippocampus is decreased when chronic epilepsy develops. This is also often the case during epileptogenesis [5]. 

The stage of epileptogenesis has also been analyzed in terms of the expression of a number of genes. Interestingly, the genes responsible for the control of many signaling pathways undergo substantial alterations in their expression. For example, the genes for mTOR (mammalian target of rapamycin), insulin-like growth factor 1, transforming growth factor β, p38MAPK (p38 mitogen-activated protein kinases) and Jak-STAT (Janus kinases-signal transducer and activator of transcription proteins) may be given [6].

## 3. AEDs—Do They Modify Epileptogenesis?

AEDs have been in clinical use for decades, and their efficacy in inhibiting seizure activity in circa 65–70% of patients has been confirmed [7]. Notably, apart from patients surviving traumatic brain injury or stroke or those with initial febrile seizures, their probable antiepileptogenic potential in clinical conditions has not been studied. As for traumatic brain injury, the preventive use of carbamazepine, phenobarbital, phenytoin or valproate to stop the development of epilepsy has failed [8]. The same holds true for diazepam and phenobarbital in cases with febrile seizures and phenytoin or valproate in patients with brain tumors [8]. On the other hand, the prospects for their antiepileptogenic activity have been evaluated in a number of animal models—post-status epilepticus models induced by pilocarpine, kainate or electrical stimulations of discrete brain areas (for instance, the amygdala and hippocampus) seem to yield data possessing a predictive potential [9].

A fundamental question arises whether AEDs may be given to patients who are at risk of epilepsy, i.e., after status epilepticus [2] or brain infection [10]. A preventive treatment with AEDs could be implemented in vulnerable patients only, where markers of epileptogenesis tend to appear. This has been best illustrated in the case of EEG markers in epileptic seizures following cerebral malaria; around 10% of children presenting the markers developed epilepsy [10]. 

The neuroprotective potential of AEDs against status epilepticus-induced brain damage in rodents has been widely documented (for review, Reference [2]). For instance, diazepam (in the low- and high-dose ranges), gabapentin, pregabalin, topiramate or valproate protected the vulnerable brain areas in animals that survived status epilepticus. There are also AEDs whose neuroprotective potential is not significant, and good examples are carbamazepine or phenytoin. As already stated above, neurodegeneration is encountered during the process of epileptogenesis, so the possibility exists that neuroprotective AEDs may at least reduce its intensity, which would eventually positively affect the frequency and intensity of seizures. In animal post-status epilepticus models, the best probable antiepileptogenic effect would be the total blockade of seizure activity following the silent period after status epilepticus. In control animals, distinct spontaneous seizure activity develops. AEDs were generally administered for a couple of days or weeks after status epilepticus—there are also examples of much longer AED administration (117 days for phenobarbital). The obtained results are mainly discouraging. Although valproate given at high daily doses for 40 days after kainate-induced status epilepticus in rats prevented hippocampal neurodegeneration and totally inhibited spontaneous seizures [2], the results obtained by other groups of investigators were the opposite. Following status epilepticus induced by pilocarpine in rats, valproate was given at 600 mg/kg daily for 3 weeks, and no neuroprotection or inhibition of spontaneous convulsions were recorded [2]. The results reported by a third group concerned status epilepticus produced by electrical stimulation of the basal amygdala in rats. Although valproate (at 600 mg/kg for 4 weeks) exerted clear-cut neuroprotection in the hippocampal area, no protection against spontaneous seizures was observed [2]. Some protective effects of gabapentin (a reduction of acquired epilepsy) or pregabalin (an extended latency to the onset of spontaneous convulsions) after chemically induced status epilepticus were noted [2]. Many conventional or newer AEDs were totally ineffective in this respect. Interestingly, diazepam at a high dose of 20 mg/kg administered as a single injection 2 h after status epilepticus evoked by electrical stimulation of the amygdala significantly reduced the number of rats exhibiting spontaneous convulsions [2]. Additionally, in the lithium-pilocarpine-induced status epilepticus in female rats, phenobarbital was administered i.p. for 2 weeks at 15 mg/kg twice daily, and then, the spontaneous seizure activity was studied for 7 days between 8 and 9 weeks after status epilepticus. Not only a reduction in the number of rats with spontaneous seizure activity was observed but the median frequency of convulsions sharply diminished from 7.5 to 1 seizure per week [11]. Another positive example might be levetiracetam (a ligand of synaptic vehicle protein SV2A [2]), however, when administered at a very high dose of 500 mg/kg (p.o. twice daily) for 4 weeks in mice after pilocarpine-produced status epilepticus [12]. The seizure activity was assessed in the presence of levetiracetam within four weeks after status epilepticus. In these circumstances, levetiracetam effectively reduced the number of spontaneous seizures, mortality and exerted neuroprotective effects [12].

Although voltage-operated sodium channel inhibitors (carbamazepine and phenytoin) seem ineffective in terms of epileptogenesis (for review, Reference [2]), a third-generation AED, eslicarbazepine, apart from the sodium channel blockade is also an effective blocker of T-type voltage-dependent calcium channels [13]. Interestingly, this drug proved an efficient inhibitor of epileptogenesis in the pilocarpine model of status epilepticus in mice. The drug was given i.p. at 150 and 300 mg/kg once daily for 6 weeks. Eventually, the spontaneous seizure activity was significantly reduced when evaluated 8 weeks after the status and the mossy fiber sprouting was considerably inhibited [14]. Thus, as a target for antiepileptogenesis, T-type voltage-dependent calcium channels could be considered. The beneficial effects of eslicarbazepine in this respect seem to support this assumption [14]. However, more preclinical studies are required on this issue. Remarkably, one other T-type calcium channel inhibitor, ethosuximide [13] at 25 and 50 mg/kg, was in the same experimental approach devoid of an antiepileptogenic activity (see below, Reference [14]).

Lamotrigine is a relatively new AED blocking voltage-operated sodium channels [13]. There are also data available suggesting the blockade of AMPA glutamate receptors at pharmacologically relevant concentrations [15], although recent data by Fukushima et al. [16] pointed to a blockade of NMDA glutamate receptors. When given i.p. 24 h after lithium-pilocarpine status epilepticus in rats at 10 or 20 mg/kg daily for one week, lamotrigine very distinctly inhibited the post-status epilepticus spontaneous seizure activity evaluated in weeks 5 and 6. In the hippocampus, neurodegeneration and astrogliosis were reduced by a lamotrigine pretreatment [17]. Ethosuximide (a T-type calcium channel blocker [13]) at 25 and 50 mg/kg in the same experimental conditions proved completely ineffective [17].

## 4. Approved Non-AEDs with an Antiepileptogenic Potential

Employing approved non-AEDs, showing a potent antiepileptogenic potency, would reduce the time necessary for implementing a novel antiepileptogenic drug compared to completely new compounds, requiring full approval procedures. No doubt, such drugs are available, and losartan seems a very promising one. Interestingly, this antihypertensive drug (a blocker of angiotensin II type 1 receptors) was evaluated in a rat model of acquired epilepsy in which sodium deoxycholate, via a craniotomy window, was administered to the brain surface. This procedure resulted in an extravasation of albumins into the brain due to vascular injury [18]. The extravasation of albumin complexes into the brain tissue may be encountered in a stroke, head trauma or infection [19,20,21]. This process is responsible for neuroinflammation involving TGF-β signaling and subsequent epileptiform activity [22]. Losartan at 100-mg/kg i.p. was administered 40 min following deoxycholate, and its relevant plasma concentration was maintained through drinking water (2 g/L) for 3 weeks. The spontaneous seizure activity was evaluated for 2 weeks, starting from the 7th day after losartan was stopped. It turned out that the losartan treatment effectively affected epileptogenesis, which was reflected by a significant reduction of rats exhibiting spontaneous convulsions. While all control rats presented clear-cut seizure activity, 60% of the animals receiving losartan were seizure-free. A sharp reduction in the average number of convulsions was also recorded, with 8/week in the control group vs. 2.25/week in the losartan group [18]. The authors carried out an additional experiment in the absence of blood–brain barrier damage. Toward this aim, they perfused albumin over the brain tissue and started to record the electrocorticographic activity for 110 days. At least two spontaneous seizures (appearing 2 days after albumin exposure) were noted in 85% of rats, and the average number of seizures reached 6.08 per week. In contrast, the proportion of seizing rats in the group perfused with albumin + losartan was reduced to 25%, the average number of seizures being 0.23 seizures/week. 

The antiepileptogenic activity of losartan was also reported in the kainate post-status epilepticus-induced epileptogenesis model in rats [23]. The angiotensin receptor 1 blocker was initiated s.c. at 10 mg/kg 2 h after the onset of status epilepticus and continued up to the 3rd day, and then, the animals were switched to losartan in drinking water up to 4 weeks. Spontaneous seizure activity was evaluated for 3 months. Apart from seizures, possible behavioral deficits and hippocampal neurodegeneration were also taken into consideration. Evidently, a pretreatment with losartan increased the latency of the onset of seizures and provided distinct neuroprotection to the CA1 hippocampal subfield. In other hippocampal regions, neuroprotection was also observed, although less expressed. Importantly, in a number of behavioral tests, losartan pretreated rats exhibited considerably less deficits [23]. When losartan was evaluated in an identical experimental approach in spontaneously hypertensive rats, the only difference observed was associated with the behavioral deficits not affected by the losartan pretreatment [24]. Some antiepileptogenic activity of losartan was confirmed in amygdala-kindled rats, because this antihypertensive drug extended the latency time of the development of fully kindled seizures [25]. The drug was either administered i.c.v. or peripherally and significantly elevated the number of stimulations needed to obtain fully kindled rats. After all, no damage to the blood/brain barrier was observed in these animals. However, some seizure parameters (threshold for after discharge induction, after discharge duration or seizure severity in fully kindled animals) were not modified by losartan pretreatment [25].

Rapamycin (an immunosuppressant drug) has been documented to block mTOR complex 1 (mammalian target of rapamycin), which is a serine/threonine protein kinase responsible for neuronal protein synthesis [26]. This drug has been tested for its potential antiepileptogenic properties in rats subjected to status epilepticus induced by kainate at 10 mg/kg [27]. In rats given rapamycin at 6 mg/kg every 4 days, a considerable reduction in the number of spontaneous convulsions was evident on days 17 and 21 after status epilepticus. Following status epilepticus produced by the electrical stimulation of the rat angular bundle, rapamycin (6 mg/kg daily for a week and then every other day for 6 weeks following status epilepticus) totally inhibited the occurrence of spontaneous seizure activity in 25% of the animals. In the remaining 75%, a significant reduction of seizure activity was shown [28]. This antiepileptogenic activity of rapamycin was associated with its neuroprotective effect in the hippocampus and a reduction in the increased permeability of the blood/brain barrier. Nevertheless, inflammation markers in the hippocampus were not affected by the pretreatment with rapamycin [28]. However, there are also data available on the lack of antiepileptogenic activity of this mTOR blocker. After 24 h following pilocarpine-induced status epilepticus in mice, the rapamycin administration (10 mg/kg) was started and continued up to 2 months. The monitoring of spontaneous motor seizures began 1 month after status epilepticus and was carried out for a month, and after 2 months, mossy fiber sprouting was evaluated. Whilst mossy fiber sprouting and dentate gyrus hypertrophy were suppressed in mice receiving rapamycin, no difference in the seizure frequency was found. Additionally, the loss of hilar neurons was not prevented. The seizure frequency reached 0.137 seizures per hour in the control group and 0.133 seizures per hour in the rapamycin group. There was a large number of subjects in both groups (N = 64) [29]. In a very similar experimental approach, rapamycin was given in a lower dose of 3 mg/kg in mice [30]. No effect of rapamycin on the spontaneous seizure frequency was noted, however, mossy fiber sprouting was reduced by 42% and the hypertrophy of the dentate gyrus was decreased compared with the control group. Nevertheless, in rapamycin-treated mice, the generation of ectopic granule cells, loss of hilar neurons or granule cell proliferation were still observed [30]. 

Potential antiepileptogenic agents may be searched for among nonsteroidal anti-inflammatory drugs affecting diverse inflammatory pathways. A good example is celecoxib, blocking the cyclooxygenase 2 and HMGB1/TLR-4 pathways, which was evaluated in rats surviving lithium-pilocarpine status epilepticus [31]. The drug was started at 20 mg/kg p.o. one day after status epilepticus and stopped at day 28th, thus covering the latent period. The parameters of spontaneous convulsions (frequency and duration observed between 28 and 42 days) were considerably reduced by the celecoxib pretreatment. Concomitantly, a potent neuroprotection was observed in the hippocampus with aberrant neurogenesis/gliogenesis being significantly decreased [31].

N-acetylcysteine is a drug approved by the FDA for the management of liver toxicity resulting from an overdose of acetaminophen, and it may also be applied as an agent loosening the thick mucus encountered in patients with chronic obstructive lung diseases [32]. The main antioxidant activity of this drug is due to its chemical structure of a reduced glutathione precursor [32]. When given an i.v. dose of 30 mg/kg immediately after systemic kainate-induced status epilepticus in rats, an increased seizure threshold of flurothyl ether was noted 12 weeks later, and mossy fiber sprouting was inhibited [33]. In another experiment, N-acetylcysteine was supplemented at 100 mg/kg p.o. for 5 weeks following brain trauma induced in rats by fluid percussion injury. Rats supplemented with the antioxidant did not react to the subthreshold dose of pentylenetetrazol (30 mg/kg i.p.), which, in control rats, induced generalized tonic-clonic seizures. Moreover, the latency of the first myoclonic jerk was considerably reduced in the vehicle-treated group, whilst the pretreatment with N-acetylcysteine brought it back to the control value of the rats without brain injury [34].

Minocycline (a second-generation tetracycline with anti-inflammatory and antioxidant properties [35]) was shown to possess some antiepileptogenic potential in lithium-pilocarpine-induced status epilepticus in rats. The authors of this study [36] provided evidence that the status epilepticus produced a prolonged activation of both astrocytes and microglia. Minocycline was given at 45 mg/kg for 2 weeks after the status epilepticus. Then, after a period of 6 weeks after minocycline was withdrawn, spontaneous recurrent convulsions were monitored for 2 weeks. The seizure activity was considerably suppressed in minocycline-pretreated animals in terms of its frequency, severity and duration. Further, this drug also mitigated the activation of microglia and reduced the elevated production of tumor necrosis factor-α and interleukin-1β in the hippocampal CA1 subfield and the neighboring cortex. However, the activation of astrocytes was not affected by the minocycline pretreatment [36]. When the status epilepticus was induced electrically in rats, minocycline failed to modify the spontaneous recurrent seizures [37]. Nevertheless, the pretreatment with this drug abolished the spatial memory deficit and normalized locomotion. No anti-inflammatory effects of minocycline were shown [37]. 

## 5. Combined Treatments

Considering a number of diverse mechanisms present during epileptogenesis, some authors have represented the point of view that combinations of drugs or agents sharing complementary mechanisms might be especially useful in this regard. The first attempt assumed there were beneficial effects of two combinations with AEDs, i.e., levetiracetam + topiramate and levetiracetam + phenobarbital, in mice after intrahippocampal kainate [38]. The former combination of levetiracetam (200 mg/kg i.p.) and topiramate (30 mg/kg i.p.) was given for 5 days (latent period), starting after 6 h from the status induction. Video/EEG monitoring for 1 week was brought about 4 and 12 weeks after intrahippocampal kainate. Brain histology was evaluated 6 and 12 weeks later. The authors distinguished electrographic seizures (observed in the EEG) and electroclinical seizures (recorded both in the EEG and videos), the former being more frequent. The combined treatment was very effective in that it reduced the frequency of spontaneous recurrent electroclinical seizures by 80% when compared to nontreated mice. The number of animals with spontaneous electroclinical convulsions was also diminished, indicating that some mice were completely protected. Moreover, the severity of the electroclinical convulsions was distinctly reduced, as manifested by less-generalized seizures (stages 4 and 5 according to a Racine scale). However, the electrographic seizure activity was not affected by the combined treatment. Moreover, no effects on the neurodegeneration or inflammatory reactions were found. The second drug combination (phenobarbital, initiated by a bolus dose of 25 mg/kg and then 3 times daily at 15 mg/kg i.p.) + levetiracetam (other details of the experiment identical to the first combination) did not modify any parameter evaluated in this study [38]. In the same model of status epilepticus in mice, a number of combinations, consisting of two to four different drugs, was evaluated in a comparable experimental approach [39]. The most beneficial combination of levetiracetam (60 mg/kg i.p.) + atorvastatin (3 mg/kg i.p.) + ceftriaxone (60 mg/kg i.p.) inhibited not only the incidence of electroclinical seizures (by 100%) but the incidence of electrographic seizures (by 60%) as well. Hippocampal neurodegeneration was not affected [39].

## 6. Antioxidative Dietary Supplements

Resveratrol (a natural phytoalexin polyphenol that may be extracted from grapes and other food products) possesses a strong antioxidative potential [40]. Apart from its antioxidative properties, the compound also exhibits anti-inflammatory and anticarcinogenic activities [41]. These mechanisms of action may be quite encouraging in terms of epileptogenesis inhibition, considering that not only inflammatory processes but also oxidative stress may be involved in this process [40]. Indeed, resveratrol proved effective in inhibiting spontaneous seizures after intrahippocampal kainate-induced status epilepticus in rats [42]. Whilst, in the control group, 75% (*N* = 12) of the rats exhibited seizure activity, only 14.3% (*N* = 7) did so in the resveratrol group. Additionally, in the latter group, a considerable reduction in the number of spontaneous recurrent seizures was shown. As regards the histological evaluation, the resveratrol group presented neuroprotection in some hippocampal areas (CA1 and CA3a), and mossy fiber sprouting was distinctly inhibited [42]. This antioxidative compound also extended the seizure latency and reduced the seizure score in pentylenetetrazol-kindled convulsions in rats, along with the neuroprotection and inhibition of oxidative stress induced by seizure activity [43].

Another compound, curcumin (an active component of turmeric), has been documented in vitro to possess, apart from an antioxidant activity, anti-inflammatory and neuroprotective properties [44]. Interestingly, the in vitro activity was not confirmed in the hippocampal tissue one week after status epilepticus in the post-electrical rat model for temporal lobe epilepsy [44]. Nevertheless, when applied during the silent period to rats following kainate-induced status epilepticus, while it did not prevent the development, it did reduce the severity of the subsequent spontaneous convulsions and offered a significant protection against cognitive impairment [45]. 

Bioactive phytochemical sulforaphane (available in broccoli sprout supplements) is an activator of the transcription factor (nuclear factor erythroid 2-related factor 2; Nrf2) responsible for the stimulation of various cellular defense lines through a number of cytoprotective genes [46]. Pauletti et al. [47] induced status epilepticus in rats via the electrical stimulation of the ventral hippocampus and then applied a combination of sulforaphane (5 mg/kg i.p.) with N-acetylcysteine (500 mg/kg i.p.) for seven days, followed by sulforaphane alone for another 7 days. Evidently, the combined treatment effectively inhibited the epileptogenesis progression as measured between 2 and 5 months, which was reflected by a significant reduction in the frequency of spontaneous seizures evaluated 5 months after the status epilepticus. Moreover, in rats receiving both antioxidants, the hippocampal neurodegeneration was considerably less expressed, and they performed better in behavioral tests for cognition. Last, but not least, oxidative stress accompanying epileptogenesis led to an expression of a neuroinflammatory molecule, the so-called high mobility group box 1 (HMBG1). A reduced post-status epilepticus oxidative stress by the two antioxidants significantly prevented the generation of HMBG1, which could be involved in the beneficial effects of the combination [47]. 

## 7. Examples of New, As-Yet Nonapproved Compounds with Antiepileptogenic Potential

As already mentioned above, rapamycin is an antagonist of the mTOR complex 1 pathway, so a question arises whether a blockade of two mTOR pathways may offer a more efficient inhibition of epileptogenesis. Towards this aim, a 1,3,5-triazine derivative (PQR620) was evaluated in mice surviving status epilepticus induced by intrahippocampal kainate [48]. PQR620 was given for 2 weeks (at doses blocking the mTOR signaling), and the seizure evaluation began 6 weeks after the drug administration was stopped. Surprisingly, no protective effect of the pretreatment with this compound on the spontaneous seizure incidence or frequency was observed. Additionally, no desired influence on granule cell dispersion in the dentate gyrus was recorded. A significant anxiety reduction was the only behavioral response seen in pretreated mice. In the same experimental approach, another triazine derivative, PQR530 (an inhibitor of the phosphoinositide-3 kinase (PI3K)-AKT/mTOR pathway), exerted an activity similar to PQR620 [48]. 

Z-944, a selective and potent antagonist of the T-type calcium channel, has been examined in rats with kainate-induced status epilepticus and subsequent epileptogenesis [49]. Z944 was administered via a continuous subcutaneous infusion at 60 mg/kg daily for 4 weeks. After a 4-week interval, the animals were tested for the occurrence of spontaneous recurrent convulsions for the next 2 weeks and behavioral abnormalities (anxiety, depression and cognition). Apparently, the group receiving the calcium channel antagonist showed less intense seizure activity manifested by a reduced number of convulsions—0.8 seizures per day (vehicle-treated rats) vs. 0.01 seizures daily. Additionally, vehicle-treated rats exhibited significant deficits in spatial learning and memory tasks, as well as distinct depressive-like behavior vs. animals without status epilepticus. The pretreatment with Z944 considerably prevented the occurrence of abnormal behaviors [49]. 

A very intriguing hypothesis regarding the role of DNA methylation was put forward by Williams-Karnesky et al. [50], who administered adenosine at 250 ng daily (for 10 days) through silk-based polymer implants into rat brain ventricles. The administration of adenosine started 9 weeks following systemic kainate (12 mg/kg, i.p.)-induced status epilepticus. Spontaneous seizures were evaluated between 10–13 and 18–21 weeks since the induction of status epilepticus. Adenosine very potently inhibited the increase in seizure frequency per week between 10 and 13 weeks and completely blocked any further increases in this parameter when evaluated between 18 and 21 weeks. Moreover, mossy fiber sprouting was significantly reduced when studied 12 weeks post-status epilepticus. According to the authors, the observed seizure-modifying effect was distinctly correlated with the inhibition of hippocampal DNA methylation [50].

Very promising effects were reported with the use of an antagomir (an antisense oligonucleotide) that targets microRNA-134 (ANT-134). Experiments were performed on the mice that survived intra-amygdalar kainate-induced status epilepticus. ANT-134 (30 mg/kg) was administered i.p. 2 h after the induction of status epilepticus, and EEG/spontaneous recurrent convulsions were recorded for 2 weeks and for 1 week after 1, 2 and 3 months following the status epilepticus. Evidently, spontaneous seizures were almost totally blocked by a single injection of ANT-134. Additionally, a reduced astrogliosis was found in the hippocampus [51]. 

Brain-derived neurotrophic factor (BDNF) is a neurotrophin involved in the modulation of synaptic plasticity and may be engaged in various functions of the central nervous system—for instance, memory processes [52]. Seizure activity is responsible for the enhanced activation of the BDNF receptor, which is tropomyosin-related kinase B (TrkB), coupled to phospholipase-C-gamma-1 [53,54]. Consequently, there is a possibility that the inhibition of TrkB signaling might be of importance in the suppression of epileptogenesis. A combined chemical–genetic approach was elaborated in order to inhibit TrkB. In wild-type mice, the enzyme is not susceptible to 1-(1,1-dimethylethyl)-3-(1-naphthalenylmethyl)-1H-pyrazolo[3,4-d]pyrimidin-4-amine (1NMPP1). However, 1NMPP1 turns into an active inhibitor after a genetic modification in the TrkB locus, which consists of the substitution of alanine for phenylalanine at residue 616. The inhibitory activity of 1NMMP1 was subsequently validated in vivo in the genetically modified mice [54]. The inhibitor was given i.p. at 16.6 μg/g 40 and 60 min after intra-amygdalar kainate (with diazepam at 10 mg/kg and lorazepam at 6 mg/kg, respectively, to terminate the status epilepticus) and then once daily. Additionally, it was available in the drinking water (at 25 μM). After 2 weeks, 1NMMP1 was withdrawn. Spontaneous seizures were measured daily in weeks 5 and 6 and behavioral testing after 8 weeks post-status epilepticus. There was a sharp reduction in the occurrence of spontaneous seizure activity in the long-term period after status epilepticus. Anxiety-like behavior was also ameliorated by the pretreatment with 1NMMP1 in genetically modified mice in the light–dark emergence test. As expected, 1NMMP1 remained ineffective in unmodified animals [54].

A similar experimental approach was used in amygdala-kindled genetically modified mice [55]. Amygdala-kindled mice (genetically modified as in the former experiment) after 6 days of a seizure-free period received an electrical stimulus inducing a seizure response (Seizure #1) and then, after 8 days, a second stimulus (Seizure #2), showing a significant progression in the seizure duration. The treatment with 1NMMP1 (16.6 μg/g) was administered i.p. after Seizure #1 every 12 h up to a total of five injections, and the mice also had access to drinking water with the TrkB inhibitor (at 25 μM) for 2 days. It turned out that, in the genetically modified mice receiving 1NMMP1, there was a clear-cut prevention of a 50% increase in the electrographic seizure duration and a 25% increase in the behavioral seizure duration, as well as a 75% increase in the duration of the ictal and postictal events. These beneficial events were not seen in the modified mice injected with the 1NMMP1 vehicle or in naïve mice administered the TrkB inhibitor itself. There is also a possibility of inhibiting TrkB signaling by the peptide pY816, uncoupling TrkB from phospholipase-C-gamma-1. Indeed, when applied after Seizure #1 at 20 mg/kg i.p. for a total of five injections in naïve amygdala-kindled mice, it very significantly prevented an increase of the seizure parameters after the induction of Seizure #2. Remarkably, carbamazepine (a conventional AED) given i.p. at 20 mg/kg every 4 h for 2 days after Seizure #1 totally failed to modify the progression of seizure activity observed at Seizure #2 [55].

## 8. c-Fos: A Potential Target for Antiepileptic Treatment

The stimulation of neurons by a variety of factors activates a group of immediate early genes (IEG), of which *c-fos* coding for the 37.5-kDa protein c-Fos is the one responding most rapidly [56,57]. Accordingly, epileptic seizures induced in experimental animals by electrical stimulation (electroconvulsive seizures, (ECS) [58,59]), or by a variety of chemicals (kainate, pilocarpine or pentylenetetrazol), are invariably accompanied by a rapid increase in the expression of c-Fos mRNA and/or proteins in the neurons of seizure-vulnerable brain regions (within minutes to a few hours following the induction of the first seizure) [60,61,62,63,64,65,66]. c-Fos activation is transient, very often receding in the latent, asymptomatic stage of epilepsy, way before the onset of recurrent seizures. Notably, in rats subjected to acute ECS, the increase of c-Fos is followed by a decrease to below the control level in the period when seizures become chronic [58]. Rapid but transient c-Fos induction has also been observed in human temporal lobe slices obtained from the surgical treatment of TLE following their epileptogenic stimulation in vitro [67].

**Figure 1:**
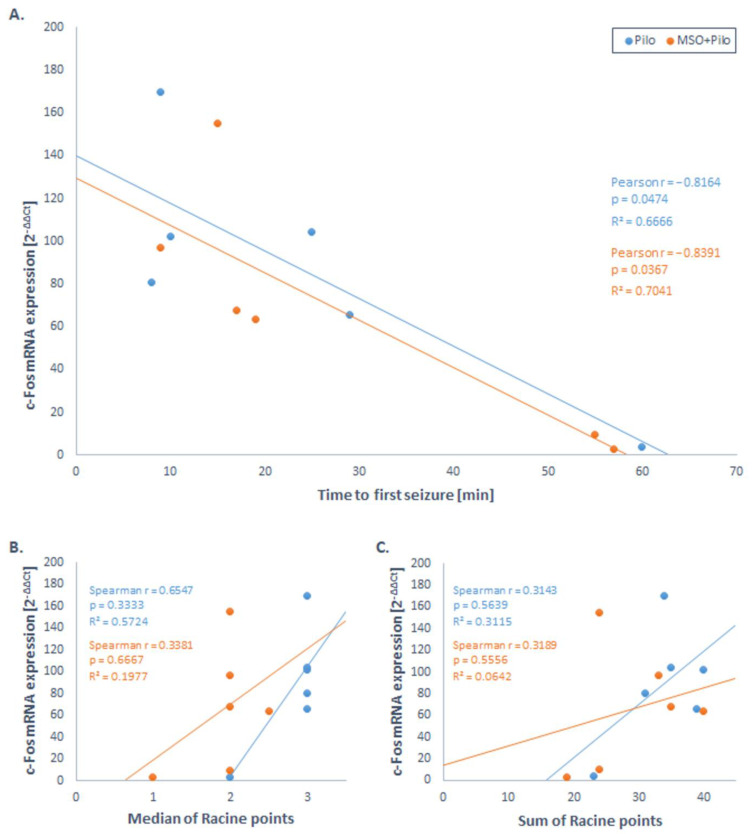
C-Fos mRNA expression in the hippocampus vs. time to the onset of the first generalized seizure (**A**) and the seizure intensity: median of the Racine points (**B**) and sum of the Racine points (**C**). Rats received one dose of glutamine synthetase inhibitor MSO (MSO + Pilo group, *N* = 6) or an equal volume of saline (Pilo group, *N* = 6) 2.5 h before the convulsive agent—pilocarpine. (**A**) Point 0 is the time of pilocarpine application. Assessment of the Racine score was made every 5 min up to 60 min after pilocarpine, when the animals were decapitated, and the brain tissue was dissected. A correlation coefficient was considered statistically significant at the two‒tailed *p*‒value <0.05. For a more detailed description of the model used, see Reference [68]. The total RNA was isolated from the hippocampus using a TRI reagent (Sigma). The extracted RNA was reverse-transcribed using the High-Capacity cDNA Reverse Transcription Kit (Applied Biosystems, Foster City, CA, USA). The mRNA expression was determined by Taqman Gene Expression Assays (Applied Biosystems) using 1 μL of cDNA in a reaction of 10 μL. The assay IDs were Rn02396759_m1 for rat c-Fos and Rn00667869_m1 for β-actin. The fold change in the gene expression was determined by the 2^−^^ΔΔCt^ method [69].

We attempted to evaluate the so-far never analyzed relation of c-Fos expression to two characteristics of initial seizures: (i) the time lapse between the stimulus application and the onset of the first seizure and (ii) seizure intensity as measured with the Racine score. In this laboratory, each of the two parameters and their correlation with glutamatergic transmission were investigated in the lithium-pilocarpine model in young rats, until 60 min after the stimulus application in a minute timescale [68]. The study revealed considerable animal-to-animal variations with regards to parameters (i) and (ii). The variability was further accentuated in a separate group of animals treated with a seizure onset-delaying glutamatergic intervener, MSO. An analysis of the brain tissue samples derived from lithium-pilocarpine animals revealed in both the MSO-treated and nontreated group a strong negative correlation of c-Fos mRNA expression with a timelapse from the pilocarpine application to the onset of the first generalized seizure but no statistically significant correlation with the seizure intensity (Figure 1). The results suggested that, the longer the animal remains resistant to the seizure-inducing stimulus, the lesser will its c-Fos response. To express this another way, the longer the animal suffers from seizures, the higher its brain c-Fos expression.

While the above observations strongly support the status of c-Fos as an early, seizure duration-related marker of epileptogenesis, they leave open the question of the contribution of c-Fos to epileptogenesis and, thus, of its assignment to the list of therapeutic targets. Since c-Fos positively regulates several aspects of neural plasticity [68], its activation in the initial stages of epileptogenesis may be considered as a neuroprotective response. Some experimental data appear to support this view. The increase of c-Fos expression elicited by trigeminal nerve stimulation coincided with, and likely contributed to, the attenuation of pentylenetetrazol-induced seizures in rats [70]. Other than in epilepsy-prone Wistar rats, in Guyenne spiny rats (*Proechimys guyannensis*), pilocarpine-induced seizures never evolve to status epilepticus; the unusual epilepsy resistance of this species is correlated with a persistent high level of c-Fos expression after the ictal stimulus [71]. However, the transient activation of c-Fos may, in most instances, be too short-lasting to mitigate a palpable degree of progression of epileptogenesis to chronic epilepsy. A c-Fos deficit at later stages of epileptogenesis could contribute to impaired cognition and memory, the well-documented associates of advanced epilepsy [72,73]. 

## 9. Conclusions

Analyzing the antiepileptogenic efficacy of AEDs and other agents, it is possible to delineate the most effective targets and targets playing a much lesser role in this regard. Considering AEDs, there are examples of their antiepileptogenic potential.

As regards the mechanisms of action of AEDs, they interact with the main three targets in the central nervous system: voltage-gated sodium or calcium channels, GABA_A_ receptor-mediated inhibition and glutamate-induced excitatory events [8,13]. The GABA_A_ receptor may, in part, appear to be a recommendable target for antiepileptogenesis, because diazepam (a positive GABA_A_ receptor modulator) at a single but high dose of 20 mg/kg very potently inhibited spontaneous seizure activity following status epilepticus induced in rats by electrical stimulation of the amygdala [74]. Valproate, an AED with multiple mechanisms of action, is also closely associated with GABA-ergic neurotransmission in that it increases GABA turnover in brain regions responsible for seizure generation and propagation [75]. However, only some, but not all, experimental data confirmed the antiepileptogenic properties of this drug (see above). Apparently, a number of factors (a method of status epilepticus induction, administration time and dosing) may influence its final antiepileptogenic effect. After all, phenobarbital, positively modulating GABA_A_ receptor-mediated inhibition [13], was shown to possess a seizure modifying the activity in rats after status epilepticus produced by lithium-pilocarpine [11]. However, phenobarbital was shown ineffective in animals in another model of status epilepticus, even when combined with levetiracetam [38].

A number of antioxidants have shown antiepileptogenic potential, so the question arises whether targeting the mechanism of the antioxidative defense might be of importance for the inhibition of epileptogenesis. Resveratrol was shown to exert diverse antioxidative effects—for instance, it reduces the production of free radicals and increases the activity of antioxidative enzymes: superoxide dismutase, catalase and glutathione peroxidase [41]. Certainly, its antioxidative properties are closely related to its anti-inflammatory activity, because free radicals have been found to promote inflammation [76]. Actually, resveratrol is an efficient suppressor of microglia-induced neuroinflammation and subsequent neuronal damage of inflammatory origin. Possibly, its anti-inflammatory activity may result from its direct inhibitory effect upon the synthesis of anti-inflammatory factors [41]. Sulforaphane was also effective in epileptogenesis inhibition, and its antioxidative activity is associated with Nrf2, which promotes the transcription of a number of antioxidant response genes [46]. Actually, sulforaphane has been shown to increase the expression of Nrf2 in the nucleus in vivo, and by the way, it also reduces the secretion of proinflammatory cytokines, which speaks to its direct interaction with nuclear factor kappa B [77]. N-acetylcysteine, exerting antiepileptogenic activity in combination with sulforaphane, is a direct antioxidant [78]. It is of importance that there are other examples of already approved drugs that share, among other properties, an antioxidative potential—for instance, minocycline [35]. This drug also exhibits an anti-inflammatory activity that is also shared by some antioxidative agents (resveratrol and sulforaphane). Curcumin is also a good example of an antioxidant and anti-inflammatory agents, as it can also act as an inflammasome silencer [79]. Summing up, the antiepileptogenic effects exerted by antioxidative compounds/drugs indicate that free radicals and antioxidant enzymes may become encouraging targets for antiepileptogenesis.

The anti-inflammatory drug celecoxib (an inhibitor of the cyclooxygenase 2 and HMGB1/TLR-4 pathways) has been documented to significantly reduce the remote consequences of status epilepticus in rats. These are recurrent spontaneous seizures, hippocampal neurodegeneration with aberrant neurogenesis/gliogenesis [31]. The beneficial antiepileptogenic activity of celecoxib may be interpreted in terms of targeting the inflammatory pathways. 

A growing body of evidence seems to suggest that the m-TOR complex may be involved in an epilepsy-modifying effect. This may be attributed to the fact that rapamycin in some post-status epilepticus models of epileptogenesis exerted a positive activity [27,28]. However, in some experimental approaches, rapamycin was completely inactive [29,30]. Probably, these discrepancies may be partially explained by the pharmacokinetics of the drug. According to Abs et al. [80], rapamycin evidently accumulates in the rat brain following its withdrawal, with still almost 50% of the drug concentration observed during continuous rapamycin administration. Thus, it is quite possible that the drug itself may be present in the brain when spontaneous seizure activity starts. The ineffectiveness of another antagonist of two mTOR pathways, PQR620 [48], speaks rather against the involvement of these pathways in epileptogenesis inhibition. 

The question arises whether the T-type calcium channel may be considered as a target for antiepileptogenesis. Indeed, the results by Casillas-Espinosa et al. [49] seemed to support such a possibility, as the potent antagonist of this channel, Z944, proved very effective in a post-status epilepticus model. Furthermore, this antagonist also very distinctly inhibited the progression of amygdala kindling in rats. Only one out of seven rats was fully kindled [81]. Interestingly, ethosuximide (also a T-type calcium channel antagonist [13]) was totally inactive in this respect—all the rats pretreated with this AED were fully kindled [81]. Perhaps the interaction mode of Z944 and ethosuximide with T-type calcium channels may vary, which can account for the completely different effects of either drug on epileptogenesis. A study by Tringham et al. [82] indicated that there were obvious differences in the mechanisms of antiseizure action of these drugs against thalamic burst firing. 

A number of microRNAs may differ in their expression in patients with neurologic diseases, and a good example is microRNA-129-2-3p, which was found elevated both in cortical tissue and the plasma of patients with temporal lobe epilepsy [83]. Recently, the antagomir-induced inhibition of microRNA-129-2-3p has been documented to efficiently block the downregulation of the *gabra1* gene, which encodes receptor subunit α_1_ of the GABA_A_ receptor complex both in rat primary hippocampal neurons and in rats with kainate-induced seizure activity [84]. Additionally, the silencing of microRNA-134 in mice surviving intra-amygdalar kainate-induced status epilepticus provided distinct neuroprotection and suppressed spontaneous seizure activity recorded between weeks 3 and 4 and 7 and 8 post-status epilepticus. The suppression of spontaneous seizure activity might be, according to the authors, dependent on the reduction by antagomir-134 of the hippocampal CA_3_ dendritic spine density [85]. Moreover, a clear-cut antiepileptogenic effect was shown above for the antagomir targeting microRNA-134 [51] after its peripheral administration. The systemic effectiveness of this compound was possible because of the blood/brain barrier disruption by kainate-induced status epilepticus [51].

Regarding c-Fos, it appears reasonable to propose an experimental antiepileptic therapy based on its extending persistence in the brain. Attempts towards this end could make use of one (or both) of the following two mechanisms:

Stimulus-induced c-Fos expression in the brain is prompted by an enhanced acetylation of histone H4 [86,87]. A wide spectrum histone deacetylase (HDAC) inhibitor, sodium butyrate, abrogated c-Fos accumulation in the resting brain [87] and attenuated epileptogenesis in the rat kindling model of TLE [88]. Moreover, an in vitro study revealed HDAC inhibition to be a common feature of three different AEDs: valproate, topiramate and levetiracetam [89], suggesting the possibility that the therapeutic effects of these drugs may be partly mediated by inhibition of c-Fos. Therefore, HDAC inhibitors specifically designed to target H4 may in the future become attractive AEDs. It has recently been postulated that HDAC inhibitors may alleviate epileptogenesis also by interacting with non-histone targets [90].

Elimination of c-Fos upon prolonged stimulation of pertinent brain regions in disease models other than epilepsy is associated with its increased interaction with a transcription factor ΔFosB, which is activated in a chronic but not in the early-stage post-stimulation [91,92]. If this mechanism applies to epilepsy, attempts at inactivating ΔFosB in due course may become a plausible therapeutic option as well.

As already pointed out above, DNA methylation or TrkB receptor-mediated events may be also considered as potential targets for the inhibition of epileptogenesis. Some clinical studies on the inhibition of epileptogenesis were conducted in patients suffering from head traumas, however, the preventive use of AEDs failed to stop posttraumatic epilepsy (for review, [2]). Specifically, carbamazepine, phenobarbital, and phenytoin were not effective at all and valproate even tended to elevate mortality in patients with posttraumatic epilepsy. There is some hope with levetiracetam which exerted a slight, albeit statistically insignificant preventive activity in this respect, [2]. Some newer clinical data on this issue are less optimistic [93]. So far, apart from AEDs, only biperiden (an anti-parkinsonian anticholinergic drug) has been evaluated in patients after brain injury in a small phase II safety assessment, prior to a double blind, randomized, placebo-controlled trial. The initial assessment confirmed its safety in patients with brain injuries and preliminary data seem to indicate that the drug seems to reduce the incidence of post-traumatic epilepsy [94]. Interestingly, biperiden has shown clear-cut antiepileptogenic properties in post-SE (induced by pilocarpine in rats) model of recurrent spontaneous convulsions [95]. Other non-antiepileptic drugs shown above, have not been tested yet in clinical trials aimed at counteracting epileptogenesis.

All the above discussed ligands considered for use as molecular targets for antiepileptogenesis are listed in Table 1. While each of them bears a clinical antiepileptogenic potential, its practical applicability can only be verified in appropriate clinical trials. Whilst patients with brain traumas may be easily recruited for such trials, patients with presumed epileptogenesis following other initial insults would have to be checked with reliable markers for epileptogenesis. Apart from already mentioned EEG markers [10], a possibility exists that also miRNAs may become reliable markers for the process of epileptogenesis or chronic epilepsy [96] and according to the here presented data—c-Fos may be also taken into consideration in this regard.

## Figures and Tables

**Table 1:** Anti-epileptogenic potential of various drugs and agents.


Anti-epileptogenic Compound			Mechanism of Action [13]							Model of Epileptogenesis	Duration of Treatment	Dosage	Effect	References
			Sodium Channel Blockade	Calcium Channel Blockade	Potassium Channel Activation	Increase in GABA Level	Increased GABA Transmission	Inhibition of Glutamate Excitation	Other Mechanisms of Action					
**Antiepileptic drugs**	**Phenobarbital**		+			+	+	+	+	Lithium-pilocarpine-induced status epilepticus in rats	14 days Spontaneous seizure activity was studied for 7 days between 8 and 9 weeks after status epilepticus	15 mg/kg/ twice daily i.p.	Reduction in the number of rats with spontaneous seizure activity; the median frequency of convulsions sharply diminished from 7.5 to 1 seizure/week	[11]
	**Levetiracetam**			+			+		+	Pilocarpine-produced status epilepticus in mice	28 days Seizure activity was assessed in the presence of levetiracetam within four weeks after status epilepticus	500 mg/kg/ twice daily, p.o.	Reduction the number of spontaneous seizures and mortality; Present neuroprotective effect	[12]
	**Eslicarbazepine**		+	+						Pilocarpine-produced status epilepticus in mice	42 days	150 and 300 mg/kg/ once daily, i.p.	Reduction spontaneous seizure activity; The mossy fiber sprouting was considerably inhibited; Inhibitor of epileptogenesis	[14]
	**Lamotrigine**		+	+	+	+		+		Lithium-pilocarpine-induced status epilepticus in rats	7 days	10 or 20 mg/kg daily, i.p. AED given 24 hours after status epilepticus	Inhibition spontaneous seizure activity; Reduction neurodegeneration and astrogliosis in the hippocampus	[17]
	**Ethosuximide**			+						Lithium-pilocarpine-induced status epilepticus in rats	7 days	25 and 50 mg/kg/daily AED given i.p. 24 hours after status epilepticus	No effect	[17]
		**Losartan**	A blocker of angiotensin II type 1 receptors							Rat model of required epilepsy in which sodium deoxycholate, via a craniotomy window, was administered to the brain surface	7 days Spontaneous seizure activity was evaluated for 2 weeks Recording electrocortico-graphic activity for 110 days	100 mg /kg i.p. Administered 40 min following deoxycholate	Significant reduction of spontaneous convulsions, sharp reduction in the average number of convulsions	[18]
										Kainate post-status epilepticus-induced epileptogenesis in rats	4 weeks Spontaneous seizure activity was evaluated for 3 months	S.c. at 10 mg/kg 2 hours after the onset of status epilepticus and continued up to the 3^rd^ day and then the animals were switched to losartan in drinking water	Increased the latency to the onset of seizures and provided distinct neuroprotection to CA1 hippocampal subfield, extended the latency time to the development of fully kindled seizures, neuroprotection being less expressed,in behavioral tests-considerably less deficits Some seizure parameters (threshold for after discharge induction, after discharge duration or seizure severity in fully kindled animals) were not modified	[23]
	**Approved non-AEDs with an anti-epileptogenic potential** **g**	**Rapamycin**	Blockade of mTOR complex 1 pathway							Kainate-induced status epilepticus in rats	21 days	6 mg/kg every 4 days	Considerable reduction in the number of spontaneous convulsions	[27]
										Electrical stimulation of the rat angular bundle	6 weeks	6 mg/kg daily for a week and then, every other day for 6 weeks following status epilepticus	Totally inhibited the occurrence of spontaneous seizure activity in 25% of animals, the remaining 75%, a significant reduction of seizure activity	[28]
										Pilocarpine-induced status epilepticus in mice	2 months	10 mg/kg	No difference in seizure frequency, Mossy fiber sprouting and dentate gyrus hypertrophy were suppressed	[29]
												3 mg/kg	Mossy fiber sprouting was reduced, hypertrophy of the dentate gyrus was decreased, generation of ectopic granule cells, loss of hilar neurons or granule cell proliferation were observed	[30]
		**Celecoxib**	Blocking cyclooxygenase 2 and HMGB1/TLR-4 pathways							Lithium-pilocarpine - induced status epilepticus in rats	28 days Parameters of spontaneous convulsions (frequency and duration) observed between 28 and 42 days	20 mg/kg p.o	Parameters of spontaneous convulsions were considerably reduced, potent neuroprotection was observed in the hippocampus with aberrant neurogenesis/gliogenesis being significantly decreased	[31]
		**N-acetylcysteine**	Chemical structure of a reduced glutathione precursor							Systemic kainate-induced status epilepticus in rats	12 weeks	I. v. at 30 mg/kg	Increased seizure threshold to flurothyl ether, the mossy fiber sprouting was inhibited	[33]
										Brain trauma in rats induced by fluid percussion injury	5 weeks	100 mg/kg p.o.	Rats supplemented with the antioxidant did not react to the subthreshold dose of pentylenetetrazol (30 mg/kg i.p.) Which in control rats induced generalized tonic-clonic seizures The latency to the first myoclonic jerk was considerably reduced in the vehicle-treated group whilst pretreatment with N-acetylcysteine brought it back to the control value of rats without brain injury	[34]
		**Minocycline**	A second generation tetracycline (with anti-inflammatory and antioxidative activity)							Lithium-pilocarpine-induced status epilepticus in rats	2 weeks Spontaneous recurrent convulsions were monitored for 2 more weeks	45 mg/kg	Seizure activity was considerably suppressed in terms of its frequency, severity and duration, activation of astrocytes was not affected	[33,35]
**Non-antiepileptic drugs/substances**	**Antioxidative dietary supplements**	**Resveratrol**	A phytoalexin polyphenol							Intrahippocampal kainate-induced status epilepticus in rats	10 days	15 mg/kg	Considerable reduction in the number of spontaneous recurrent seizures was shown, neuroprotection in some hippocampal areas (CA1 and CA3a) and mossy fiber sprouting was distinctly inhibited	[40]
										Pentylenetetrazol-kindled convulsions in rats	10 weeks	25mg/kg, 50mg/kg and 75 mg/kg	Reduced the seizure score in pentylenetetrazol-kindled convulsions in rats, along with neuroprotection and inhibition of oxidative stress	[41]
		**Curcumin**	Antioxidant activity, also anti-inflammatory and neuroprotective properties							Post-electrical rat model for temporal lobe epilepsy	1 week	2 μl of 2 mm curcumin solution	In vitro activity was not confirmed in hippocampal tissue	[42]
										Kainate-induced status epilepticus in rats	14 days	100 mg/kg	Did not prevent the development but reduced the severity of subsequent spontaneous convulsions and offered a significant protection against cognitive impairment	[43]
		**Sulforaphane**	An activator of the transcription factor (nuclear factor erythroid 2-related factor 2; Nrf2) responsible for the stimulation of various cellular defense lines through a number of cytoprotective genes							Induced status epilepticus in rats via electrical stimulation of the ventral hippocampus	7 days + 7 days Epileptogenesis progression Measured between 2 and 5 months	Sulforaphane (5 mg/kg i.p.) With N-acetylcysteine (500 mg/kg i.p.) For seven days followed by sulforaphane alone for another 7 days	Inhibited epileptogenesis progression which was reflected by the significant reduction in the frequency of spontaneous seizures evaluated 5 months after status epilepticus, Neurodegeneration was considerably less expressed and they performed better in behavioral tests for cognition	[45]
	Examples of compounds with anti-epileptogenic potential PQR530 Z-944 Adenosine Antagomir	**PQR620**	Blockade of mTOR pathways							Status epilepticus induced by intrahippocamal kainate	2 weeks The seizure evaluation begun 6 weeks after the drug administration was stopped	At doses blocking the mTOR signaling	No protective effect of the pretreatment with this compound on spontaneous seizure incidence or frequency was observe, no influence on granule cell dispersion in the dentate gyrus was recorded	[46]
		**PQR530**	An inhibitor of the phosphoinositide-3 kinase (PI3K)-AKT/mTOR pathway)											
		**Z-944**	Selective and potent antagonists of T-type calcium channel							Kainate-induced status epilepticus and subsequent epileptogenesis in rats	4 weeks The animals were tested for the occurrence of spontaneous recurrent convulsions for the next 2 weeks	60 mg/kg daily via continuous subcutaneous infusion	Less intense seizure activity manifested by reduced number of convulsions, significant deficits in spatial learning and memory task as well as distinct depressive-like behavior vs. Animals without status epilepticus	[47]
		**Adenosine**	Induces hypomethylation of DNA via biochemical interference with the transmethylation pathway							Systemic kainate -induced status epilepticus in rats	10 days Spontaneous seizures were evaluated between 10-13 and 18-21 weeks since the induction of status epilepticus.	250 ng daily through silk-based polymer implant into rat brain ventricles	Potently inhibited the increase in seizure frequency per week between 10-13 weeks and completely blocked the further increase in this parameter when evaluated between 18-21 weeks, mossy fiber sprouting was significantly reduced when studied 12 weeks post status epilepticus	[48]
		**Antagomir**	An antisense oligonucleotide which targets microRNA-134 (ANT-134)							Intraamygdalar kainate-induced status epilepticus in rats	2 hours after the induction of status epilepticus	30 mg/kg, i.p.	Spontaneous seizures were almost totally blocked by a the single injection, reduced astrogliosis was found in the hippocampus	[49]
		**1NMMP1**	1NMPP1 turns into an active inhibitor after a genetic modification in the trkb (BDNF receptor which is tropomyosin-related kinase B) locus which consists in a substitution of alanine for phenylalanine at residue 616.							Amygdalar kainate-induced status epilepticus in mice	5-6 weeks - measuring spontaneous seizures After 8 weeks post-status epilepticus - behavioral testing	16.6 μg/g ,40 and 60 min after intra-amygdalar kainate (with diazepam at 10 mg/kg and lorazepam at 6 mg/kg), respectively, to terminate status epilepticus) and then once daily. Also available in the drinking water (at 25 μm). After 2 weeks, 1NMMP1 was withdrawn.	Sharp reduction in the occurrence of spontaneous seizure activity in the long-term period after status epileptics, anxiety-like behavior was ameliorated in genetically modified mice in the light-dark emergence test, remained ineffective in unmodified animals	[52]
										Amygdala-kindled mice 6 days of seizure free period received an electrical stimulus inducing a seizure response (Seizure #1) and then, after 8 days – a second stimulus (Seizure #2)	8 days	16.6 μg/g i.p. After the seizure #1 every 12 hours up to a total of 5 injections and the mice also had access to the drinking water with the trkb inhibitor (at 25 μm) for 2 days. Applied after Seizure #1 at 20 mg/kg i.p. For a total of 5 injections in naïve amygdala-kindled mice	In genetically modified mice there was a clear cut prevention of the 50% increase in electrographic seizure duration, 25% increase in behavioral seizure duration as well as 75% increase in duration of ictal and postictal events. Beneficial events were not seen in modified mice or in naïve mice administered the TrkB inhibitor itself	[53]
Combined treatments		Levetiracetam		+			+		+	Intrahippocampal kainate-induced status epilepticus in mice	5 days (latent period), starting after 6 hours from the status induction	Levetiracetam (200 mg/kg i.p.) + topiramate (30 mg/kg i.p.)	Reduction the frequency of spontaneous recurrent electroclinical seizures by 80% when compared to non-treated mice and generalized seizures (stages 4 and 5 according to a Racine scale). Reduction a number of animals with spontaneous electroclinical convulsions (some mice were completely protected); No effects on electrographic seizure activity, neurodegeneration or inflammatory reactions	[54]
		**Topiramate**	+	+	+	+	+	+	+					
		Levetiracetam		+			+		+	Intrahippocamal kainate-induced status epilepticus in mice	5 days (latent period), starting after 6 hours from the status induction	Phenobarbital, initiated by a bolus dose of 25 mg/kg and then 3 times daily at 15 mg/kg i.p.) + levetiracetam (200 mg/kg i.p.)	No effect on incidence or frequency of electroclinical or electrographic seizures	[54]
		**Phenobarbital**	**+**			+	+	+	+					
		Levetiracetam		+			+		+	Intrahippocamal kainate-induced status epilepticus in mice	5 days (latent period), starting after 6 hours from the status induction	Levetiracetam (60 mg/kg i.p.) + atorvastatin (3 mg/kg i.p.) + ceftriaxone (60 mg/kg i.p.)	Reduce the incidence of electroclinical seizures (by 100%) and the incidence of electrographic seizures (by 60%) as well; No effect on hippocampal neurodegeneration	[55]
		**Atorvastatin**	A competitive inhibitor of HMG-coa reductase [55]											
		**Ceftriaxone**	“reverses posttraumatic downregulation of glutamate transport in the brain” [55]											

## Data Availability

Not applicable.
